# Warwick-Edinburgh Mental Well-being Scale (WEMWBS): Validated for teenage school students in England and Scotland. A mixed methods assessment

**DOI:** 10.1186/1471-2458-11-487

**Published:** 2011-06-21

**Authors:** Aileen Clarke, Tim Friede, Rebecca Putz, Jacquie Ashdown, Steven Martin, Amy Blake, Yaser Adi, Jane Parkinson, Pamela Flynn, Stephen Platt, Sarah Stewart-Brown

**Affiliations:** 1 Health Sciences Research Institute, Warwick Medical School, University of Warwick, Coventry, CV4 7AL, UK; 2 University Medical Center, Göttingen, Dept. of Medical Statistics, 37073 Göttingen, Germany; 3 NHS Birmingham East and North,4th Floor, Waterlinks House Richard Street.Aston, Birmingham, B7 4AA, UK; 4 The Caludon Centre, Coventry and Warwickshire Partnership Trust, Clifford Bridge Road, Coventry CV2 2TE, UK; 5 College of Medicine, King Saud University; Shaikh Abdullah Bahamdan Research Chair for Evidence-Based Health Care and Knowledge Translation; P.O.Box 2925, Riyadh 11461, Saudi Arabia; 6 NHS Health Scotland, Elphinstone House, 65 West Regent Street, Glasgow G2 2AF, UK; 7 Department of Sports Studies, University of Stirling, Stirling, FK9 4LA, UK; 8 Centre for Population Health Sciences, University of Edinburgh, Teviot Place, Edinburgh EH8 9AG, UK

## Abstract

**Background:**

Understanding and measuring mental health and wellbeing amongst teenagers has recently become a priority. The Warwick-Edinburgh Mental Well-being Scale (WEMWBS) is validated for measuring mental wellbeing in populations aged 16 years and over in the UK. We report here a study designed to establish the validity and reliability of WEMWBS in teenagers in the UK.

**Methods:**

WEMWBS and comparator scales, together with socio-demographic information and self-reported health, were incorporated into a self-administered questionnaire given to pupils aged 13 to 16 years in six schools in Scotland and England. Psychometric properties including internal consistency, correlations with comparator scales, test-retest stability and unidimensionality were investigated for WEMWBS. Twelve focus groups were undertaken to assess acceptability and comprehensibility of WEMWBS and were taped, transcribed and analysed thematically.

**Results:**

A total of 1,650 teenagers completed the questionnaire (response rate 80.8%). Mean WEMWBS score was 48.8 (SD 6.8; median 49). Response scores covered the full range (from 14 to 70). WEMWBS demonstrated strong internal consistency and a high Cronbach's alpha of 0.87 (95% CI (0.85-0.88), n = 1517). Measures of construct validity gave values as predicted. The correlation coefficient for WEMWBS total score and psychological wellbeing domain of the Kidscreen-27 was 0.59 (95% CI [0.55; 0.62]); for the Mental Health Continuum Short Form (MHC-SF) was 0.65, 95% CI [0.62; 0.69]; and for the WHO (WHO-5) Well-being Index 0.57 (95% CI [0.53; 0.61]). The correlation coefficient for the Strengths and Difficulties Questionnaire (SDQ) was -0.44 (95% CI [-0.49; -0.40]) and for the 12-item General Health Questionnaire (GHQ12) -0.45 (95% CI [-0.49; -0.40]). Test-retest reliability was acceptable (Intraclass correlation coefficient (ICC) 0.66 (95% CI [0.59; 0.72] n = 212)). Confirmatory factor analysis demonstrated one underlying factor.

WEMWBS was significantly associated with the Family Affluence Score (WEMWBS increased with increasing household socio-economic status) and had a positive association with the physical health dimension of the Kidscreen-27, but was unrelated to age, gender or location/school. Eighty students took part in focus groups. In general, although some students considered some items open to misunderstanding or misinterpretation, WEMWBS was received positively and was considered comprehensible, and acceptable.

**Conclusions:**

WEMWBS is a psychometrically strong population measure of mental wellbeing, and can be used for this purpose in teenagers aged 13 and over.

## Background

During the past 25 years, emotional and behavioural problems have been recognised as a major cause of ill health among adolescents in industrialised nations in addition to physical complaints [1-3]. Such problems are associated with an increased likelihood of poor later life outcomes such as depression [4], impaired social relationships [5] and substance misuse[6,7], as well as increased financial costs at the societal level due to criminal behaviour, extra educational support and social care [8].

As a result, the prevention of emotional and behavioural problems and the promotion of emotional, and social and mental wellbeing is now a national priority for children and adolescents in England. Its importance has been emphasised in "Every Child Matters," [9] the National Service Framework for Children, [10] guidance from the UK National Institute for Health and Clinical Excellence [11] and in recommended indicators to assess school performance by the England Office for Standards in Education (Ofsted) [12]. Improving children's and young people's mental wellbeing is also a national priority in Scotland, [13] and in many other countries in Europe.

However, whilst the definition of mental illness in childhood and adolescence has been the subject of much investigation, definitions of mental wellbeing are less well researched. In adults, mental wellbeing is regarded as covering both hedonic (happiness, subjective wellbeing) and eudaimonic (positive functioning) wellbeing [14]. In 2004, the Royal Society in the UK defined wellbeing as a "*positive and sustainable *mental state that allows individuals, groups and nations to *thrive and flourish.". *Mental wellbeing, conceptualised as more than the absence of mental illness, appears protective for a range of health outcomes including self-rated general health [15], immunity response, stress response [16] and predictors of cardiovascular disease survival [17,18]. Mental wellbeing has also been found to be associated with higher educational attainment in childhood and adolescence, and with better occupational functioning in adulthood [1-3].

This increasing focus on mental wellbeing in teenagers and adolescents has created the need for validated scales to monitor teenage population mental wellbeing and evaluate interventions and programmes targeted at this age group. The Warwick-Edinburgh Mental Well-being Scale (WEMWBS), which was developed to provide a measure of mental wellbeing suitable for use in the UK adult population, presented as a possible candidate. Key attributes of WEMWBS were its focus on the positive, its face validity among the general population, public health practitioners and policy makers in the UK, and its normal distribution in the general population with no floor or ceiling effects. WEMWBS is based on the Affectometer 2, a measure developed in New Zealand in the 1980's [19] and was designed following literature review, and UK validation, incorporating the views of a multidisciplinary expert panel [20,21]. WEMWBS covers both eudaimonic and hedonic aspects of wellbeing as does the Affectometer 2.

WEMWBS has been found easy to complete, clear and unambiguous in research conducted with adult focus groups [21] and has proved popular with practitioners and policy makers both in the UK and further afield [20,21]. We report here findings from a study which aimed to assess the validity and reliability of the WEMWBS in a population sample of school students aged 13/14 and 15/16 years in six schools in two cities in England and Scotland in 2008-2009 [22].

## Methods

### Questionnaire design and scales used

A questionnaire was designed and piloted. It included the WEMWBS, socio-demographic variables (age and ethnicity as recorded in the UK Census) and the Family Affluence Scale (FAS) [23] which is a child-friendly measure of family socioeconomic status (scores range from 0 to 7, higher scores indicate greater affluence). The theoretical range of scores for WEMWBS is 14-70, with higher scores indicating a higher level of mental wellbeing. Comparator scales included three scales to measure mental wellbeing:

• The World Health Organisation WHO-5 Well-being Index (WHO-5) [24] (scores in the range of 0 (worst outcome) to 100 (best outcome));

• The Kidscreen-27 scale [25] with five standardised domain scores with mean 50 and standard deviation 10 (higher scores indicate better health states);

• The Mental Health Continuum-Short Form (MHC-SF) [26,27] summarised as total sum ranging from 0 to 70 (higher scores indicate greater flourishing) and as three categories "languishing", "moderately mentally healthy," and "flourishing."

Two scales were used to measure mental health problems:

• General Health Questionnaire 12 item version (GHQ12) [28] summarised as item total score (range 0-12) and total sum based on Likert scoring (range 0-36) (higher scores indicate poorer mental health);

• Strengths and Difficulties Questionnaire (SDQ) [29,30] summarised as a total difficulties score which can range from 0 to 40 (lower scores indicate fewer difficulties).

All these scales apart from the MHC-SF have been validated for use in adolescents in the UK.

### Study conduct

Ethics committee approval was obtained and included "opt-out" consent for parents/carers. Six schools (3 each from two cities, one in Scotland and one in England) were selected to reflect variation by geographical location, socioeconomic deprivation (based on proportion of children in receipt of free school meals) and educational attainment (proportion of children achieving 5+ GCSE grades A-C (England)/*5+ *awards at *SCQF *Level 4 (Scotland). Approval for participation was sought from local authority education departments and head teachers, and letters explaining the purpose and procedures of the study were sent to parents/carers, teachers and students. Students in two year groups (years 9 and 11 in England and S2 and S4 in Scotland (aged 13/14 and 15/16 years)) in each school completed questionnaires during lesson times. All data were double entered. In order to assess test-retest reliability, a randomly selected 12% sample of participants completed the WEMWBS on a second occasion, between 7 and 14 days after initial administration of the questionnaire.

Twelve focus groups, (six in each city, two in each school) were conducted with students from the same schools and year groups but who were not involved in the questionnaire survey. Groups lasted 45-60 minutes and comprised 6-8 students of the same age and gender, selected to reflect a range of academic ability. Focus groups were chosen both for convenience and because we considered that the effect of group dynamics might promote a wider discussion of the issues raised. Discussions were held in a separate private room by researchers. All students completed the WEMWBS scale, and a protocol was used to structure subsequent discussion. All discussions were tape recorded and transcribed (with consent) and NVivo [31] was used to manage the data. Initial analyses were undertaken concurrently with data collection to confirm that data saturation had been achieved.

### Psychometric analysis

Descriptive statistics (including means and standard deviations) were calculated for all scales and subscales using SAS and rules for dealing with missing items were followed in each case. Frequencies and percentages were computed for categorical scores. Associations between WEMWBS and socio-demographic variables were investigated in linear regression models, with WEMWBS as the dependent variable and socio-demographic variables as independent variables. Factors statistically significant at the 10% level in univariate analyses were included simultaneously in a multiple linear regression model.

Associations between physical health and socioeconomic status with WEMWBS score were assessed using Kidscreen-27 physical wellbeing score and the FAS respectively, using first univariate then multiple regression analyses. Internal consistency of WEMWBS was assessed using Cronbach's alpha [32]. Spearman's rank correlation coefficients and Pearson correlation coefficients were calculated for each item with the total of the remaining items. Confidence intervals were obtained by nonparametric bootstrapping with 9,999 bootstrap replications [33]. For the correlational analysis, p-values of approximate significance tests, testing the null hypothesis of "no correlation", were calculated. Since the results were very similar only the nonparametric Spearman's coefficients are reported here.

Spearman's rank correlation coefficients (rho) were calculated for correlations of the WEMWBS scale, on the one hand and Kidscreen-27; the GHQ12, the WHO-5, MHC-SF and the SDQ, on the other, for assessment of construct validity. Again, correlation coefficients were calculated with nonparametric bootstrap confidence intervals with 9,999 bootstrap replications and p-values of approximate significance tests, testing the null hypothesis of "no correlation." We report here the Spearman rank correlation coefficient, rather than the Pearson correlation coefficient, to make results directly comparable to those presented in Tennant *et al*. [20,21] However, we also computed the Pearson coefficients and found that the differences between the two types of coefficient were generally small with the maximum difference in absolute values amounting to 0.04.

An estimate of the intraclass correlation coefficient (ICC) was obtained [34,35] with 95% confidence interval using a random effects model with individual random effects for the participants.

Confirmatory factor analysis of all 14 WEMWBS items to test the hypothesis of a one-factor structure of WEMWBS was performed. A structural equation model, with one latent factor was fitted by weighted least squares. Initially we assumed independent residuals. We subsequently added parameters allowing for pairwise dependencies between residuals in a stepwise fashion guided by analyses of the covariance structure [36].

### Qualitative analysis

Analysis was undertaken using an adapted Framework Approach [37] suitable where a framework for analysis already exists. Each focus group recording was coded thematically. Codes were based on the structured protocol used to guide the discussions and subsequently combined into over-arching themes, on the basis of repeated readings of the transcripts.

Analysis was then conducted across the whole dataset (all groups combined). Subsequently, the data were explored in order to identify systematic differences by location, gender and age group. Three researchers analysed transcripts independently, reading and re-reading them to identify emerging key themes, discrepancies, deviant cases and correspondences in the data. Convergence and divergence between the three analyses were discussed and agreed and account was taken of the differing perspectives of the contributing researchers.

## Results

### Psychometric results

Of a possible total 2042 questionnaires, 1650 were completed, giving an overall response rate of 80.8%. Response rates by school ranged from 62.1% to 91.9%. The sample was representative of schools and the underlying populations of teenagers, with approximately equal numbers of boys and girls. Participants were predominantly white (78%), with 69% scoring 5 or higher on the Family Affluence Scale (FAS), indicating that they lived in households of middle or higher affluence. Table 1 shows respondent characteristics.

**Table 1:** Respondent Characteristics

Characteristic		N (%)
Gender	Male	808 (49.1)

	Female	838 (50.1)

	Missing	4 (0.2)

Age	13 years and under ^1^	772 (46.8)

	14 years	204 (12.4)

	15 years	564 (34.2)

	16 years and over	107 (6.5)

	Missing	3 (0.18)

Ethnicity	White	1269 (78.4)

	Asian or Asian British	220 (13.6)

	Other	129 (16.0)

	Missing	32(2.0)

Family Affluence Scale	1-4	499 (30.4)

	5-7	1139 (69.4)
	Missing	12 (0.7)

^1 ^We used whole school year and class groups as our sampling frame. Ages ranged slightly outside year group standards. The majority of respondents (94.3%) was aged 13-16 years. ("13 years and younger" includes 78 twelve year olds. "16 years and over" includes eight over-16s)

**Figure 1:**
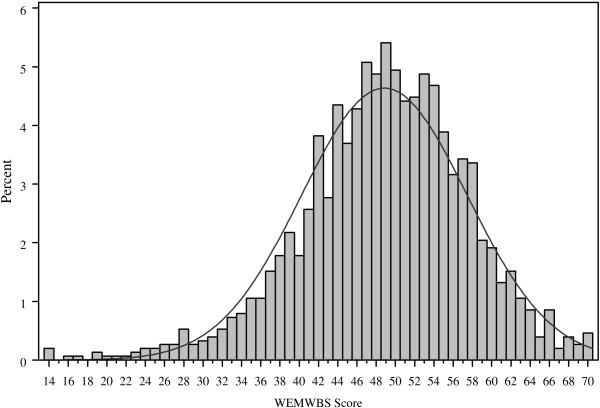
**Unadjusted WEMWBS scores (n = 1517)**.

Ninety-two percent (1,517 of 1650 participants) completed all questions in the WEMWBS questionnaire and a further 5.5% (91 participants) answered 13 of 14 items. The mean WEMWBS score was 48.8 (standard deviation (SD) 8.6) and the median was 49. WEMWBS scores ranged from 14 to 70 (see Figure 1), i.e. the full range of possible values was used, with no evident ceiling or floor effects.

Scores for boys were 1.8 points higher than for girls, but this difference disappeared after adjustment for age, FAS and physical wellbeing. Similarly after adjustment for gender and FAS, WEMWBS scores were not found to vary significantly with age.

A significant association was found between WEMWBS scores and FAS after adjustment for age, gender and physical wellbeing. A FAS score of 5+ was associated with an increase in WEMWBS of 1.47 (95% CI [0.61, 2.32]; p < 0.001) compared to a FAS score of 1-4. Adjusted WEMWBS scores did not differ significantly between schools.

**Figure 2:**
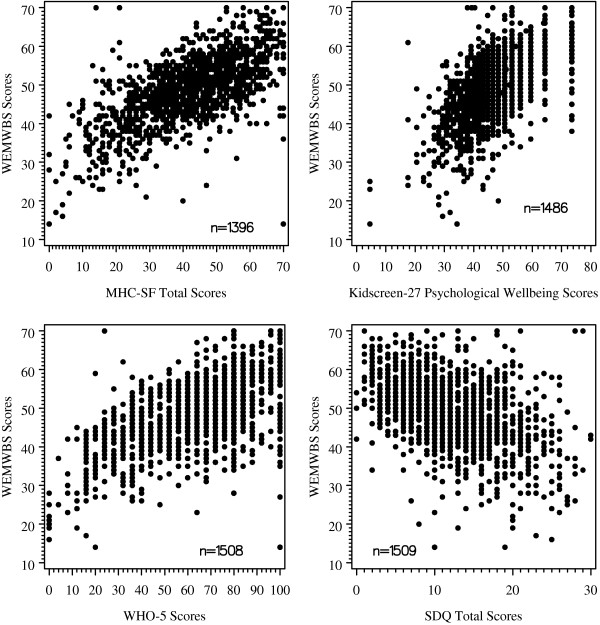
**Scatter plots: WEMWBS vs. MHC-SF (Total score: n = 1396), Kidscreen-27 (Psychological Well-being domain: n = 1486), WHO-5 (n = 1508) and SDQ (Total score: n = 1509)**.

Table 2 shows Spearman's rank correlation coefficients with 95% confidence intervals for WEMWBS scores with GHQ12, WHO-5, SDQ, MHC-SF and Kidscreen-27. Correlations with scales or subscales measuring mental wellbeing were statistically significant, of moderate strength and positive. (MHC-SF total score; rho = 0.65, 95% CI [0.62; 0.69]; psychological wellbeing domain of the Kidscreen-27 (rho = 0.59, 95% CI [0.55; 0.62]) and the WHO-5 (rho = 0.57, 95% CI [0.53; 0.61]). Correlation with scales measuring mental health problems were similarly of moderate strength and statistically significant, but negative. (SDQ total score (rho = -0.44 (95% CI [-0.49; -0.40])); GHQ12 score (rho = -0.45 (95% CI [-0.49; -0.40])). Higher WEMWBS scores were also associated with increased physical wellbeing scores. (Kidscreen-27 Physical Well-being domain scores were moderately strongly correlated with WEMWBS scores (rho = 0.43, 95% CI [0.39; 0.47])). Figure 2 illustrates some of these relationships using scatter plots (WEMWBS versus the MHC-SF total score, the psychological wellbeing domain of the Kidscreen-27, WHO-5, SDQ total score and GHQ score).

**Table 2:** Correlation coefficients for WEMWBS with GHQ12, WHO-5, SDQ and Kidscreen-27

Scale		N	Correlation coefficients (rho)	95% CI		p-value
GHQ12	Scores	1479	-.45	-.49	-.40	<0.001
	
	Likert	1479	-.52	-.56	-.47	<0.001

WHO-5		1508	0.57	0.53	0.61	<0.001

SDQ	Total	1509	-.44	-.49	-.40	<0.001

MHC-SF	Total	1396	0.65	0.62	0.69	<0.001
	
	Category	1396	0.57	0.53	0.61	<0.001

Kidscreen-27	Physical Well-being	1499	0.43	0.39	0.47	<0.001
	
	Psychological Well-being	1486	0.59	0.55	0.62	<0.001
	
	Autonomy & Parent Relation	1484	0.46	0.42	0.50	<0.001
	
	Social Support & Peers	1492	0.38	0.33	0.42	<0.001
	
	School Environment	1489	0.51	0.46	0.55	<0.001

We found very little difference between age groups when correlations between WEMWBS and GHQ12, WHO-5, SDQ, MHC-SF and Kidscreen-27 were repeated for those aged 14 years and younger versus those aged 15 years and older. (Data available on request). All correlation coefficients were within 0.05 (except for MHC Category (0.08)) and confidence intervals for all correlation coefficients overlapped, indicating no statistically significant differences by age group in the strength of correlations.

### Internal consistency

Cronbach's alpha for WEMWBS was 0.87 (95% CI [0.85; 0.88]). The lower limit of the confidence interval lies above 0.7, demonstrating satisfactory to high internal consistency [32]. Spearman's rank correlation coefficients with 95% confidence intervals were assessed for each WEMWBS item with the total of the remaining items. All WEMWBS items had correlations with the total of the remaining items which fell within the desirable range, (0.2 to 0.8). Furthermore, all confidence intervals lay entirely within the range demonstrating that all items had the desired substantial, but not excessive, correlation.

### Test-retest reliability and dimensionality - confirmatory factor analysis

Two hundred and twelve (212) test-retest pairs of completed WEMWBS questionnaires, representing 12.8% of respondents were compared giving an intra class correlation coefficient (ICC) of 0.66 (95% CI [0.59; 0.72]). The final one-factor structural equation model included 28 parameters for pairwise residual correlations and was fitted using data from 1517 students. The goodness-of-fit test resulted in a chi-square statistic of 48.74 with 48 degrees of freedom (p = 0.443), demonstrating good model fit and confirming the hypothesised one-factor structure of WEMWBS which was supported by a Goodness of Fit Index (GFI) of 1.0000, a GFI Adjusted for Degrees of Freedom (AGFI) of 0.9999 and a Root Mean Square Error of Approximation (RMSEA) of 0.0032.

### Focus group results

Eighty mixed ability students (40 aged 13-14 years and 40 aged 15-16 years) took part in single sex focus groups. Across both year groups and genders, mental wellbeing was perceived to be strongly associated with happiness and positivity, while being mentally unwell was associated with sadness, worry and depression. Table 3 shows WEMWBS with items numbered, each of which was considered in turn by focus group members. Most participants found the scale simple, short and easy to complete. Participants suggested that some individuals might find it embarrassing to complete and that some items were asking about matters which they did not often think about, for example "dealing with problems well", "thinking clearly" or having "energy to spare".

**Table 3:** Warwick-Edinburgh Mental Well-being Scale (WEMWBS)

STATEMENTS	None of the time	Rarely	Some of the time	Often	All of the time
1. I've been feeling optimistic about the future	1	2	3	4	5

2. I've been feeling useful	1	2	3	4	5

3. I've been feeling relaxed	1	2	3	4	5

4. I've been feeling interested in other people	1	2	3	4	5

5. I've had energy to spare	1	2	3	4	5

6. I've been dealing with problems well	1	2	3	4	5

7. I've been thinking clearly	1	2	3	4	5

8. I've been feeling good about myself	1	2	3	4	5
9. I've been feeling close to other people	1	2	3	4	5

10. I've been feeling confident	1	2	3	4	5

11. I've been able to make up my own mind about things	1	2	3	4	5
12. I've been feeling loved	1	2	3	4	5

13. I've been interested in new things	1	2	3	4	5

14. I've been feeling cheerful	1	2	3	4	5

Below are some statements about feelings and thoughts. Please tick the box that best describes your experience of each over the **last 2 weeks**.

"Warwick-Edinburgh Mental Wellbeing Scale (WEMWBS). ^©^NHS Health Scotland, University of Warwick and University of Edinburgh, 2006, all rights reserved."

Items 3 ("feeling relaxed"), 8 ("feeling good about myself"), 11 ("able to make up my own mind") and 14 ("feeling cheerful") were understood by all groups and thought relevant. There were some issues of definition and understanding for items 1 ("feeling optimistic") and 5 (having "energy to spare"). Items which attracted a more variable interpretation were often those where a more holistic, reflective, less "concrete" approach to oneself is needed e.g. item 2 ("feeling useful"), 6 ("dealing with problems well"), 7 ("thinking clearly"), 10 ("feeling confident") and 13 ("interested in new things"). Girls were more confident than boys in interpreting items 9 ("feeling close to other people") and 10 ("feeling confident") and boys tended to be more confident than girls in interpreting item 13 ("interested in new things"). Items which could be construed as relating to a sexual or romantic relationship (e.g. item 4 ("interested in other people" - which was often interpreted as attraction to a prospective girlfriend/boyfriend), item 12 ("feeling loved") and item 9 ("feeling close to other people")) had the potential to cause misinterpretation, hilarity or embarrassment.

While the overall length of the scale was acceptable, it was felt that there might be some redundancy which could be removed through amalgamation of items. On the other hand some participants suggested items that could be added to the scale. The setting for administration tended to confuse some participants, who contextualised their responses within the school setting rather than considering their lives as a whole. The scale was received positively. All participants said that they filled out the scale honestly, although some felt that others might not do so.

## Discussion

WEMWBS is a population measure of mental wellbeing, validated for use in adults aged 16 years and over in the UK [20,21]. We undertook a rigorous mixed-methods study of validity and reliability of WEMWBS in a large population sample of school students aged 13-16 years in Scotland and England. We found associations between physical health (using the Kidscreen-27 physical wellbeing score) and socioeconomic status (measured using the Family Affluence Scale), but no separate association between WEMWBS and age or gender once adjustment for these other factors had been made. The scale demonstrated an appropriate range of values, with very few missing items. Measures of construct validity gave values as predicted (positive correlations between WEMWBS and WHO-5, the psychological wellbeing domain of the Kidscreen-27 and the MHC-SF scale; negative correlations with the SDQ total difficulties score and GHQ12 scores). WEMWBS has a strong internal consistency in this population group, with a high Cronbach's alpha and strong internal positive correlations between individual items and total scores. The test-retest correlation was slightly lower than anticipated (ICC = 0.66 (95% CI [0.59; 0.72] n = 212)). Given the large numbers and high response rate, it is unlikely that this is a chance finding. It may reflect more day to day variation in mental wellbeing in this age group than in adults, although this would require corroborating through further research. Findings are stable at the population level for which the scale is intended.

Eighty students took part in focus groups. WEMWBS was received positively. The overall underlying construct and response categories were understood. Some participants identified some individual words or terms as either difficult to understand or open to misinterpretation and the school setting for administration of the scale tended to confuse some participants. Whilst most participants found the scale simple, short, comprehensible and easy to complete, the qualitative findings suggest that comprehension may be a problem for some adolescents and children younger than 13 years, although this effect was not apparent in the quantitative findings.

Some students experienced embarrassment in relation to items which could be construed as having a link to a sexual or romantic relationship. It is possible that this finding might have been different if we had undertaken individual interviews.

Concurrent qualitative investigation of scale face validity, of the depth undertaken in this study, is unusual, and we believe that the issues we identified are likely to be found with other scales in common usage subjected to a similarly rigorous process. Although these qualitative findings suggest that WEMWBS could be enhanced by modifying items, the loss of continuity with the adult scale which would result is a significant disincentive.

Because the school setting tended to confuse some participants, we recommend that when WEMWBS is introduced to teenagers in a school environment, its holistic nature is emphasised.

## Conclusions

The promotion of emotional, social and mental wellbeing in teenagers and adolescents is becoming a national priority in a number of countries. WEMWBS is one of the few solely positive single scales for measuring mental wellbeing which has been fully validated for use in this age group. The scale is appropriate for use in those aged 13 years to adulthood.

## Competing interests

JP is employed by the funding body for this study: NHS Health Scotland. SSB, JP and SP are authors of the original design and validation of WEMWBS. (See references 16 and 17).

## Authors' contributions

All authors contributed to, design, data collection and analysis and critically revised and approved the manuscript.

## Pre-publication history

The pre-publication history for this paper can be accessed here:

http://www.biomedcentral.com/1471-2458/11/487/prepub
